# Physical activity and influencing factors in people post stroke or transient ischemic attack across diverse regions in Sweden

**DOI:** 10.3389/fneur.2024.1463162

**Published:** 2024-10-30

**Authors:** Lucian Bezuidenhout, Sophia Humphries, David Moulaee Conradsson

**Affiliations:** ^1^Division of Physiotherapy, Department of Neurobiology, Care Sciences and Society, Karolinska Institutet, Stockholm, Sweden; ^2^Division of Physiotherapy, Department of Health and Rehabilitation Sciences, Stellenbosch University, Cape Town, South Africa; ^3^Medical Unit Occupational Therapy & Physiotherapy, Theme Women's Health and Allied Health Professional, Karolinska University Hospital, Stockholm, Sweden

**Keywords:** stroke, transient ischemic attack, physical activity, sedentary behavior, environmental factors

## Abstract

**Background and purpose:**

Physical activity (PA) and sedentary behavior are key targets for secondary stroke prevention, yet their characteristics and contributing factors are not well understood. This study aims to explore PA and sedentary behavior in individuals' post-stroke or transient ischemic attack (TIA) and identify factors linked to low PA (≤5,000 steps/day) and prolonged sedentary time (≥8 h/day).

**Methods:**

A cross-sectional study comparing sensor-derived (activPAL) PA and sedentary time among community-dwelling individuals post stroke or TIA residing in diverse geographical regions of Sweden. Multiple logistic regression models were performed to determine potential factors associated with low PA and prolonged sedentary time.

**Results:**

The study included 101 participants post-stroke (*n* = 68) and TIA (*n* = 33), with a mean age of 70.5 years (65% female), mostly with no or mild disability (91%), living in metropolitan (69%) and rural (31%) areas of Sweden. Most participants (72%) had ≥ 8 h of sedentary time per day and 38% performed ≤5,000 steps per day. Using a walking aid (OR = 11.43, *p* = 0.002) was independently associated with low PA, whereas contextual factors; living alone (OR = 3.49, *p* = 0.029) and living in metropolitan areas (OR = 2.79, *p* = 0.036), were associated with prolonged sedentary time.

**Discussion and conclusions:**

In this study encompassing people post stroke or TIA from diverse geographical regions across Sweden, PA was associated with mobility status whereas sedentary behavior was associated with contextual factors. The results also showed a large variation in PA highlighting the need for tailored strategies to promote PA post stroke or TIA.

## Introduction

Stroke is one of the leading causes of mortality and disability globally (1, 2), with mild strokes being the most prevalent type, accounting for about 60% of all strokes (3, 4). People with mild stroke or transient ischemic attack (TIA) may exhibit a significant burden of functional disability and fatigue with an increased risk of mortality remaining years after the onset of stroke or TIA (3, 5). Individuals who suffered a first mild stroke or TIA have a 26% increased risk of recurrent stroke within 5 years (6) and the second stroke is often more fatal and disabling (7). Secondary stroke prevention targets include addressing vascular risk factors, such as blood pressure control, and promoting a healthy lifestyle regarding diet, alcohol consumption, and physical activity (PA) (8).

Physical activity (i.e., any bodily movement produced by skeletal muscles that requires energy expenditure) and sedentary behavior (i.e., any waking activity characterized by an energy expenditure of ≤ 1.5 metabolic equivalents) (9) represent unique aspects of daily life and are important targets for secondary stroke prevention (10). A frequently used proxy for PA is the number of steps taken per day, while sedentary behavior is often measured by the amount of time spent sitting or lying down. It is important to distinguish between the two behaviors as recent evidence has suggested that individuals who meet the recommended guideline for PA, but still accumulate prolonged sedentary time may still face risk to their metabolic health (11). Moreover, despite sedentary behavior posing similar risks to cardiovascular health, most rehabilitation strategies focus on increasing PA levels, with less emphasis on reducing sedentary behavior (12). Physical activity has also been well recognized to be a modifiable risk factor for secondary stroke (13), where engaging in regular PA after a mild stroke or TIA reduces the 5-year risk of stroke-related disability by 44% and recurrence by 48% (5).

The simplest and most popular form of PA is walking, therefore regular ambulation at a certain intensity and duration are common goals for people post stroke or TIA (14). A recent systematic review has shown that people post stroke took on average 5,535 steps per day, well below that measured in healthy participants (8,388 steps per day) (13). In addition, low PA and increased sedentary time have been linked with elevated blood pressure and increased risk of cardiovascular diseases and all-cause mortality (15). For example, a meta-analysis found that individuals sitting for >8 h per day had a 59% higher mortality rate compared to those that were active for about 60–75 min per day of moderate intensity PA (16). A study by English et al. (1) showed that people with mild stroke spend about 75% (10.9 h/day) of their wake hours sitting. Furthermore, individuals post stroke are reported to spend significantly more time sitting (10.9 vs. 8.2 h/day) (1) compared to healthy individuals. While the growing evidence shows that PA significantly benefits overall health and lowers the risk of recurrent stroke, it is crucial to comprehend how individuals post stroke or TIA engage in PA relative to their sedentary behavior.

Previous studies (4, 17) have shown that higher age, balance impairment, low self-efficacy, and symptoms of fatigue, depression and anxiety are associated with low PA post stroke (4, 17). With regards to sedentary behavior, previous studies have shown increased body mass index, use of a walking an aid, higher stroke severity and less degree of independence in activities in daily living to be associated with greater sedentary behavior post stroke (15). It has been recognized that features of the social and physical environment could act as barriers or facilitators to PA (18, 19). However, few studies have has examined how environmental factors affect PA and sedentary time in people post stroke or TIA (20), with most studies focusing on motor and psychosocial determinants (21). Recent studies have highlighted the significance of social support (e.g., living alone) as a facilitator and the physical environment (e.g., living in a rural area as compared to an urban area) as a barrier to engagement in PA post stroke or TIA (21, 22). Additionally, most previous studies examining PA behavior and factors associated with PA post stroke have included community-dwelling people post stroke, with few investigations including those with TIA (23).

This study aimed to describe and compare sensor-derived PA levels among people post stroke or TIA with different ambulation profiles and sedentary behavior, and to determine factors associated with low physical activity (≤5,000 steps per day) and prolonged sedentary time (≥8 h of sitting time per day). By examining these behaviors separately, this study seeks to inform tailored intervention strategies for promoting PA in people post stroke or TIA.

## Materials and methods

### Study setting and ethical considerations

This cross-sectional study used baseline data from a pilot randomized controlled trial investigating the feasibility of a mobile health program for PA in people post stroke or TIA across Sweden (24). This study was approved by the Swedish Ethical Review Authority (dnr. 2020-05062 and 2021-03622). All study participants provided written informed consent before study participation.

### Participants

People post stroke or TIA were recruited through advertisements at local or regional (Stockholm) and national patient organizations, on the homepage of Karolinska Institutet and social media. The inclusion criteria were clinical diagnosis of stroke or TIA confirmed through a doctor certificate, between ≥ 3 months and 10 years before study enrolment, with the ability to walk short distances indoors with or without a walking aid. The exclusion criteria were severe health conditions (e.g., cardiac conditions, other neurological diseases, and severe arthritis), significant cognitive impairment, neglect or aphasia compromising the ability to give written consent or use a mobile application to complete digital questionaries in the study.

Participant eligibility screening involved a two-step process. First, interested individuals participated in a telephone interview to assess their eligibility based on stroke/TIA diagnosis, living conditions, ambulation status, and mobile app proficiency. Second, participants were granted access to the STAAR mobile application (Stroke Treatment through Active and Accessible Rehabilitation), which facilitated data collection and administered digital surveys for the study. A video call through the app was conducted to verify participants' ability to use the app, follow instructions, and maintain attention. Final inclusion decisions were based on this screening and a doctor's certificate confirming the diagnosis.

### Data collection

By collecting data remotely without requiring physical visits, data was collected from individuals post stroke or TIA from diverse geographical regions across Sweden. This included administering digital questionnaires through a mobile application and assessment of sensor-derived PA. Data collection was conducted during two periods (September–November 2021 and September–November 2022).

Seven digital questionnaires were administered: (1) Demographics (age, sex, level of education, living situations, geographical location of residency, and stroke or TIA diagnosis), (2) Self-perceived impact of stroke or TIA and stroke recovery assessed using the total sum score of the 8 domains and visual analog scale (VAS; 0 = no recovery to 100 = full recovery) of the Stroke Impact Scale, respectively (25). (3) Balance confidence assessed using the sum score of the Activities-Specific Balance Confidence (ABC) scale (0 = no confidence in balance to 100 = complete confidence in performing the activity) which was specifically developed for application in stroke rehabilitation (26), (4) Self-efficacy for exercises assessed using the Swedish version of Exercise for Self-Efficacy scale (0 = no confidence to 90 = completely confident), which has shown high relative reliability and internal consistency for use in measuring exercise self-efficacy in people with neurological diseases (27), (5) Walking ability assessed using the sum score of the Swedish version of the Generic Walk-12 scale (0 = no walking difficulties to 42 = more walking difficulties) (28), (6) Fatigue was assessed using the Fatigue Severity Scale (FSS), a validated scale with demonstrated reliability in people post stroke. Each item is scored on a 7-point Likert scale, ranging from 1 to 7, and the scores are summed to calculate a total mean score. A score greater than 4 indicates fatigue in stroke patients (29), and (7) Depression, anxiety and stress were assessed using the sum scores pertaining to each of the subscales of the Depression Anxiety Stress Scale (DASS) (30). The DASS is a 21-item questionnaire scored using a 4-point Likert scale (0–3). The scale is divided into three subscales, each containing seven items, with individual scoring systems for depression, anxiety, and stress. Scores range from normal (0–9 for depression, 0–7 for anxiety, and 0–14 for stress) to very serious (≥28 for depression, ≥20 for anxiety, and ≥34 for stress) for each subscale, respectively. The DASS has been recommended as a measure for depression, anxiety and stress in clinical populations due to its strong psychometric properties (30). Additionally, the Modified Rankin Scale was used to assess the degree of disability (31).

Physical activity was measured using the activPAL (PAL Technologies Ltd) activity monitor at a sampling frequency of 10 Hz. The activPAL is a small (43 × 23.5 × 5 mm) triaxial accelerometer and inclinometer worn on the thigh using waterproof tape. ActivPAL is reliable in measuring step count, time spent sitting, standing and walking time in different populations (1, 32, 33) and has been recommended for the measurement of PA and sedentary behavior post stroke (34). The monitor was posted to the participants, and they were instructed to wear the device on the non-hemiparetic leg (or dominant leg if no hemiparesis) for 7 consecutive days (24 h/day) and return the device through prepaid postal service.

### Data management

Physical activity data were downloaded as 15-second epochs, exported as.csv files using the PAL analysis software (v 8.11.8.75). The timestamp data were then used to divide the data into daily segments (midnight to midnight). Participants with ≥3 days and with ≥10 h per day of PA recordings were included in the analysis (35). Physical activity outcomes were average daily steps, walking time, sitting time, number of sit-to-stand transitions, and fragmentation index. The fragmentation index (i.e., number of sitting bouts/total sitting hours) gives insight into whether the participants accumulate their sitting time in many short bouts or a smaller number of longer bouts (36). Since individuals post stroke or TIA may have different physical capacities depending on the level of disability (37, 38), study participants were classified into two PA groups: ≤5,000 steps/day and >5,000 steps/day, and two groups based on sedentary behavior: ≥8 h spent sitting/day and < 8 h spent sitting/day (39).

### Statistical analysis

Data were analyzed using IBM SPSS Statistics (v. 24.0) software. Numbers (percentages) and mean (standard deviation) were used to present descriptive data on demographics, disability level, stroke-related variables, balance confidence, self-efficacy for exercise, walking ability, fatigue, symptoms of depression, stress and anxiety, and PA outcomes. Physical activity outcomes were tested for data normality using the Shapiro-Wilk Test and visual inspection of histograms, confirming that the data were normally distributed.

For the first aim, independent *t*-tests were used to compare the PA outcomes between groups walking ≤5,000 steps/day and >5,000 steps/day, and between groups spending ≥8 h per day sedentary and < 8 h per day sedentary.

For the second aim, two multiple logistic regression models were performed. Potential factors associated with low PA (≤5,000 steps/day, model 1) and prolonged sedentary time (≥8 h/day, model 2) were first entered one at a time in univariate logistic regression models. Table 1 outlines the factors included and the criteria for categorization used in the logistic regression model. Since there were no reference cut-off points for self-efficacy for exercise and walking ability, we used the median scores. The threshold for including factors in the multivariate logistic analyses was set to an α level *P* ≤ 0.1. Prior to multivariable analyses, measures of association between covariates were checked by variance inflation statistics and by creating a bivariate matrix for correlations. A variance inflation factor value of < 5 and a Spearman correlation coefficient of < 0.7 was used as a cut-off for the correlation between covariates (3). Factors were subsequently entered into a multivariable logistic regression model with an α level set at *P* ≤ 0.05 for the identification of independent factors associated with low PA and prolonged sedentary time.

**Table 1:** Personal and stroke-related factors with the cut-off points used for the logistic regression model.

	**Criteria for categorization**
**Personal factors**	
Age	>70 years/ ≤ 70 years
Sex	Male/female
Education	Low education (Primary and High school)/High education (University degree)
Living condition	Living alone/co-habiting
Location	Rural/metropolitan
**Stroke-related factors**	
Diagnosis	Stroke/TIA
Self-perceived recovery (visual analog scale of stroke impact scale)	< 85 = less recovery/≥85 = more recovery (52)
Walking aid	Yes/No
ABC	≤ 81% = indication of poor balance/>81% = indication of good balance (53)
Self-efficacy for exercise	≤ 65 = less self-efficacy for exercise/>65 high self-efficacy for exercise
Generic Walk-12	≥8 = more walking difficulties/ < 8 = less walking difficulties
Fatigue severity scale	≥36 = indication of moderate to severe fatigue/ < 36 = indication of mild or no fatigue (54)
DASS depression subscale	≥9 = indication of depression/ < 9 = normal or depression
DASS anxiety subscale	≥7 = indication of anxiety/ < 7 = normal or no anxiety
DASS stress subscale	≥14 = indication of stress/ < 14 = normal or no stress

## Results

### Participant characteristics

Out of the 114 participants included in the study, 13 were excluded from the analysis due to technical problems (i.e., battery failure or data downloading/transferring error) with the activPAL monitor (*n* = 7) or < 3 valid days of PA data (*n* = 6). The study characteristics of the included participants (*n* = 101) are presented in Table 2. Participants were recruited from 20 out of the 21 regions in Sweden. The average age of the participants was 71 years, and the majority had a stroke (*n* = 68, 67%), experienced mild disability (*n* = 67, 66%), and lived in a metropolitan area (*n* = 70, 69%). A minority lived alone (*n* = 38, 38%) and ambulated with a walking aid (*n* = 18, 18%).

**Table 2:** Study characteristics of people post stroke or TIA.

**Age (years), mean (SD)**	**70.5 (9.2)**
Sex male, *n* (%)	35 (35)
**Education**, ***n*** **(%)**	
Primary school	10 (10)
High school	28 (28)
University degree	63 (62)
**Employment status**, ***n*** **(%)**	
Working	17 (17)
Sick leave	10 (10)
Retired	73 (73)
Living alone, *n* (%)	38 (38)
**Location**	
Metropolitan	70 (69)
Rural	31 (31)
Stroke, *n* (%)	68 (67)
TIA, *n* (%)	33 (33)
**Comorbidities**	
Diabetes mellitus, n (%)	10 (9.9)
Hypertension, *n* (%)	61 (60.4)
Heart disease, *n* (%)	23 (22.8)
Joint disease, *n* (%)	42 (41.6)
**Modified ranking scale**, ***n*** **(%)**	
No symptoms	25 (25)
No significant disability	67 (66)
Slight disability	6 (6)
Moderate disability	2 (2)
Severe disability	1 (1)
Stroke Impact Scale, mean (SD)	36.5 (7.9)
Self-perceived recovery of stroke,^a^mean (SD)	75 (23)
Use of walking aid, *n* (%)	18 (18)
ABC, mean (SD)	78 (21)
Self-efficacy for Exercise, mean (SD)	64.4 (15.8)
Generic Walk-12 scale, mean (SD)	9.6 (8.4)
Fatique severity scale, mean (SD)	35.0 (12.4)
DASS depression subscale, mean (SD)	6.5 (7.0)
DASS anxiety subscale, mean (SD)	3.8 (3.9)
DASS stress subscale, mean (SD)	7.4 (6.9)

TIA, transient ischemic attack; ABC, Activities-Specific Balance Confidence; DASS, Depression Anxiety Stress Scale. ^a^As measured by the visual analog score of the stroke impact scale.

### Physical activity post stroke or TIA in relation to ambulation status and sedentary behavior

**Figure 1:**
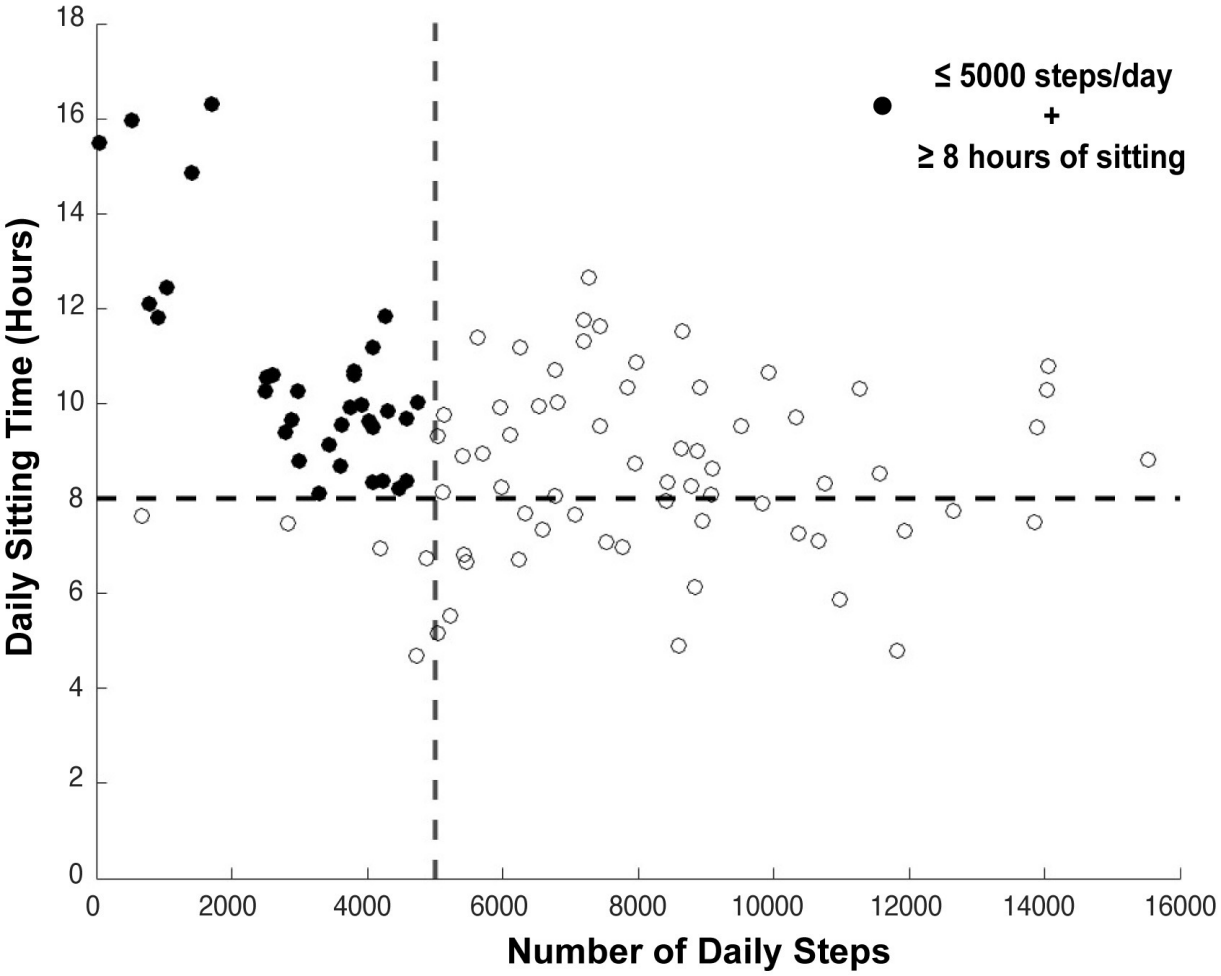
Relationship between the daily sitting time and steps for each individual post stroke. The dashed lines indicate the cut-point for physically active (>5,000 steps) and prolonged sitting (>8 h). The filled dots refer to individuals post stroke with risk behavior (i.e., ≤5,000 steps/day and ≥8 h of sitting/day) of recurrent stroke and disability in the future.

Figure 1 shows the distribution of the number of daily steps in relation to sedentary time of participants post stroke or TIA. The majority had ≥8 h of sitting/day (*n* = 73, 72%) and 4 out of 10 (*n* = 38, 38%) performed ≤5,000 steps/day. Thirty-three (33%) participants took ≤5,000 steps/day and had a sitting time ≥ 8 h, while 40 (40%) of the participants took >5,000 steps/day and had ≥ 8 h/day of sedentary time.

There was a significant difference in all PA outcomes between the groups walking ≤5,000 steps/day and >5,000 steps/day (Table 3). The group spending ≥ 8 hours/day sedentary spent less time walking (76 min vs. 102 min, *p* = 0.002), more time sitting (609 min vs. 409 min, *p* < 0.001), and had a lower fragmentation index (5.0 vs. 8.0 bouts per sitting hour, *p* < 0.001) compared to the group spending < 8 h/day sedentary.

**Table 3:** Description of physical outcomes.

**Outcomes, mean (SD)**	**All participants (*N =* 101)**	** ≤5,000 steps/day (*N =* 38)**	**>5,000 steps/day (*N =* 63)**	***P*-value**	**≥8 h sitting h/day (*N* =73)**	** < 8 h sitting h/day (*N* = 28)**	***P*-value**
Number of measured days	6.4 (0.8)	6.4 (0.9)	6.5 (0.7)	0.415	6.4 (0.7)	6.5 (0.8)	0.781
Steps/day	6,539 (3,529)	3,150 (1,361)	8,584 (2,776)	< 0.001	5,990 (3,398)	7,971 (3,523)	0.011
Daily walking time (min/day)	83.2 (38.8)	46.0 (19.2)	105.6 (29.1)	< 0.001	75.9 (37.0)	101.9 (37.6)	0.002
Sitting time (min/day)	553.2 (131.1)	605.8 (149.3)	521.4 (108.3)	0.001	608.7 (106.3)	408.5 (59.2)	< 0.001
Transitions	46.7 (13.6)	40.5 (13.0)	50.7 (10.3)	< 0.001	45.2 (12.4)	51.4 (12.3)	0.024
Fragmentation index	5.4 (2.1)	4.3 (1.9)	6.1 (1.8)	< 0.001	4.6 (1.5)	7.6 (1.7)	< 0.001

### Factors associated with low physical activity (≤5,000 steps/day)

The univariate logistic regression models showed that having a stroke (Odds Ratio (OR) = 2.46, *p* = 0.057), self-perceived recovery score of < 85 (OR = 2.81, *p* = 0.020), using a walking aid (OR = 12.6, *p* < 0.001), ABC score ≤ 81.1% (OR = 4.41, *p* < 0.001), Generic Walk-12 score ≥8 (OR = 3.39, *p* = 0.005), DASS depression score ≥9 (OR = 2.59, *p* = 0.031), and DASS stress score ≥14 (OR = 7.59, *p* = 0.003) were independently associated with walking ≤5,000 steps/day. The multivariate logistic regression model showed that using a walking aid (OR = 11.4, *p* = 0.002) was the only factor that remained significantly associated with walking ≤5,000 steps/day (Table 4).

**Table 4:** Univariate and multivariate logistic regression models for factors predicting physical inactivity and prolonged sitting.

	**Physical inactivity (**<**5,000 steps/day)**				**Prolonged sedentary behavior (**≥**8 h sitting time)**			
	**Univariate model**		**Multivariate model**		**Univariate model**		**Multivariate model**	
**Predictors**	**OR (95 % CI)**	***P*-value**	**OR (95 % CI)**	***P*-value**	**OR (95 % CI)**	***P*-value**	**OR (95 % CI)**	***P*-value**
Age, < 70 (ref) > 70	0.75 (0.33–1.69)	0.487			0.74 (0.31–1.78)	0.503		
Sex, Female (ref) Male	0.94 (0.41–2.14)	0.876			0.96 (0.40–2.31)	0.920		
Education, University (ref) Primary and High school	1.08 (0.48–2.44)	0.847			2.15 (0.85–5.45)	0.108		
Living alone, No (ref) Yes	1.76 (0.78–3.99)	0.176			4.13 (1.42–11.97)	0.009	3.49 (1.14–10.68)	0.029
Location, Rural (ref) City	0.80 (0.35–1.86)	0.608			2.45 (1.02–6.04)	0.044	2.79 (1.07–7.25)	0.036
TIA (ref) Stroke	2.46 (0.97–6.2)	0.057	0.66 (0.17–2.53)	0.515	1.43 (0.58–3.52)	0.432		
Self-perceived recovery^a^, ≥ 85 (ref) < 85	2.81 (1.18–6.73)	0.020	1.04 (0.34–3.17)	0.946	1.13 (0.47–2.73)	0.778		
Walking aid, No (ref) Yes	12.63 (4.42–36.07)	< 0.001	11.43 (2.44–53.61)	0.002	1.76 (0.63–4.92)	0.278		
ABC, > 81.% (ref) ≤ 81.1 %	4.41 (1.88–10.31)	< 0.001	1.58 (0.34–7.45)	0.562	1.72 (0.69–4.27)	0.241		
Exercise for Self-efficacy, ≥ 65 (ref) ≤ 65	1.34 (0.61–2.98)	0.466			0.85 (0.36–2.00)	0.711		
Generic Walk-12, < 8 (ref) ≥ 8	3.39 (1.44–7.97)	0.005	1.40 (0.41–4.79)	0.594	2.52 (1.03–6.15)	0.043	2.12 (0.81–5.53)	0.125
Fatigue severity, < 36 (ref) ≥ 36	1.63 (0.73–3.62)	0.230			1.27 (0.54–2.99)	0.581		
DASS depression subscale, < 9 (ref) ≥ 9	2.59 (1.09–6.14)	0.031	1.29 (0.35–4.68)	0.702	2.08 (0.75–5.76)	0.157		
DASS anxiety subscale, < 7 (ref) ≥ 7	2.28 (0.85–6.12)	0.103			2.73 (0.74–10.13)	0.133		
DASS stress subscale, < 14 (ref) ≥ 14	7.59 (1.96–29.30)	0.003	4.28 (0.69–26.50)	0.118	2.75 (0.58–13.14)	0.204		

TIA, transient ischemic attack; ABC, Activities-Specific Balance Confidence; DASS, Depression Anxiety Stress Scale. ^a^As measured by the visual analog score of the stroke impact scale.

### Factors associated with sedentary behavior (≥8 h of sitting per day)

The univariate logistic regression model showed that living alone (OR = 4.13, *p* = 0.009), living in a metropolitan geographical area (OR = 2.45, *p* = 0.044), and a Generic Walk-12 score of ≥8 (OR = 2.52, *p* = 0.043) were associated with spending ≥8 h/day sedentary. The multivariate logistic model showed that living alone (OR = 3.49, *p* = 0.029) and living in a metropolitan geographical area (OR = 2.79, *p* = 0.036) remained independently associated with sedentary behavior.

## Discussion

The results showed a large variation in PA and sedentary behavior among people post stroke or TIA across diverse regions in Sweden. About one-third of the participants walked ≤5,000 steps/day and spent ≥ 8 h/day sedentary, indicating a risk behavior of recurrent stroke and disability in the future (5). Furthermore, using a walking aid was associated with low PA post stroke or TIA, whereas contextual factors; living alone and living in metropolitan areas, were independently associated with sedentary time.

Contrary to our results, Miller et al. (21) found living alone to be associated with greater daily step counts post stroke (21). In line with the present findings, previous research has suggested that social support for PA (i.e., family members, partners) (40), comfort levels of caregivers (41) and social roles in the household (41) positively influence the PA, that leads to less sedentary behavior in people post stroke. Furthermore, our results showed that living in a metropolitan area was significantly associated with prolonged sedentary time in people post stroke or TIA. This is consistent with previous findings, which suggest that healthy adults in metropolitan areas, according to self-reported and objective data, spend more time sedentary compared to those in rural areas (42, 43). To our knowledge, no previous research has examined the relationship between physical environment (rural vs. metropolitan) and sedentary behavior in individuals post stroke. Our result highlights the need to further explore the role of the environment for promotion of PA behavior in people post stroke or TIA. Previous studies investigating factors associated with PA levels (e.g., steps/day) identified age, balance, self-efficacy, anxiety, depression, and fatigue as contributing factors post stroke/TIA (4, 17). In relation to sedentary behavior (e.g., sitting time) earlier research has indicated that age, functional independence, stroke severity, and walking speed are contributing factors post stroke/TIA (15, 44, 45). This is consistent with the univariate models in our study. However, in the multivariate model, only the use of a walking aid was significantly associated with low PA post stroke or TIA. The differences in the results could be explained by differences in stroke characteristics and the criteria for categorizing the predictors in the regression models.

People with stroke or TIA in this study who were physically active (i.e., >5,000 steps/day) engaged in a greater number of sit-to-stand transitions and fragmentation index compared to those who walked ≤5,000 steps/day. This is consistent with previous studies comparing the number of steps/day and sedentary time between people post stroke with different physical capacities (35). For example, Fini et al. (35) showed that community ambulators people post stroke with a self-selected gait speed ≥0.8 m/s took more steps/day (469 vs. 4,975) and spent less time sedentary (1,239 vs. 1,105 min) compared to those with a gait speed < 0.8 m/s (35). Therefore, the difference in PA behavior observed between the PA groups in our study could be attributed to the ability to ambulate, which directly influences their capacity to engage in daily PA. The PA level among the group walking ≤5,000 steps/day in our study, averaging 3,150 steps per day, mirrors the level of 3,786 steps/day previously identified as indicative of stroke recurrence risk in individuals with minor stroke (46). This indicates the need to increase PA of individuals with ≤5,000 steps/day to reduce the risk of recurrent stroke. Individuals post-stroke or TIA may exhibit varying PA levels based on disability levels (37, 38). Thus, understanding disparities in PA levels and sedentary behavior among subgroups can pinpoint those at elevated risk. Most existing research primarily focuses on describing PA behaviors post stroke. However, it is crucial to acknowledge that while PA and sedentary behavior are related, they are distinct constructs with different associated risks for cardiovascular disease (47). Specifically, individuals post-stroke who engage in adequate PA may still experience adverse cardiovascular effects if they accumulate prolonged periods of sedentary time. Therefore, rehabilitation strategies should not only promote meeting the recommended PA guidelines for people post stroke/TIA but also promote reducing sedentary behavior (i.e., through regular movements and frequent breaks from sitting), as outlined in the international recommendation for secondary stroke prevention (48).

Previous evidence has suggested that irrespective of their previous PA levels, people who have suffered a mild stroke or TIA make no changes to their PA behavior after the stroke onset (49). Contrary to what previous research has shown (13), the stroke participants in our study had a relatively high PA levels, which is comparable to healthy adults (13). High PA level before stroke onset has been linked to reduced stroke severity and improved long-term functional outcomes (50). However, since we have no data on participants' pre-stroke PA levels, we cannot confirm this. Our results are also in line with previous studies (1) indicating most people post stroke are sedentary. In our study, 72% of the individuals post stroke/TIA had prolonged sedentary hours, with a significant difference between the two sitting groups post stroke regarding sitting time, daily walking time and fragmentation index. Our findings also showed that 40% of participants engaged in PA (>5,000 steps/day) and had extended sedentary hours (≥8 h of sitting/day), highlighting the importance of considering PA and sedentary behavior as distinct concepts.

### Limitations

This was a cross-sectional study and therefore we cannot infer causality between independent factors and PA levels and sedentary behavior post stroke or TIA. Second, people with primarily mild stroke or TIA with the ability to ambulate indoors with or without a walking device were recruited from a clinical trial investigating the feasibility of a mobile health intervention for the promotion of PA post stroke or TIA (24). This inadvertently introduced the risk of selection bias of individuals interested in and aware of PA and health promotion. The age of the study sample was approximately 5 years younger than the population (75 years) and the proportion of women (65%) and those with a university degree (63%) was slightly higher (51). Future studies should aim to include a more diverse study sample post stroke/TIA, particularly regarding a greater representation of men, older adults, and diversity in socioeconomic status. Third, all data, except for the PA measures, were self-reported, which may affect the reliability of the outcomes, particularly in cases of cognitive impairments which is common post stroke. The digital format precludes the inclusion of performance-based clinical tests (e.g., walking, balance, and symptom representations). On the other hand, the digital format facilitated the inclusion of a diverse group of community-dwelling people post stroke or TIA from various geographical regions in Sweden with large variability in PA levels and sedentary behavior. Fourth, the use of cut-points to categorize covariates in regression models may have resulted in the decrease of the sensitivity of the data, while continuous covariates could improve model precision. Finally, possible mediation effects between certain covariates in the logistic models may exist, which may influence the significance of these covariates.

## Conclusion

In this study encompassing people post stroke or TIA from diverse geographical regions across Sweden, PA was associated with mobility status whereas sedentary behaviors were associated with contextual factors. The results also showed a large variation in PA and sedentary behavior highlighting the importance of tailored strategies for PA promotion post stroke or TIA.

## Data Availability

The datasets presented in this article are not readily available because it contains information that could compromise the privacy of the research participants. Requests to access the datasets should be directed to Research Data Office at the Karolinska Institute (rdo@ki.se).
